# Editorial: Hot Topics in Pancreatology From Europe-2020

**DOI:** 10.3389/fmed.2021.724457

**Published:** 2021-09-08

**Authors:** Gabriele Capurso, Sebastien Gaujoux, Enrique de-Madaria

**Affiliations:** ^1^Pancreato-Biliary Endoscopy and Endosonography Division, Pancreas Translational & Clinical Research Center, San Raffaele Scientific Institute Istituto di Ricovero e Cura a Carattere Scientifico, Milan, Italy; ^2^Department of Digestive, Hepatobiliary and Endocrine Surgery, Paris Descartes University, Cochin Hospital, Paris, France; ^3^Université de Paris, Paris, France; ^4^Gastroenterology Department, Alicante University General Hospital, ISABIAL, Alicante, Spain

**Keywords:** pancreatology, Pancreas 2000, mentoring, European pancreatic club, future

The study of the physiology and diseases of the exocrine pancreas is a highly vocational sub-specialty that is of interest to not only gastroenterologists and surgeons, but also radiologists, oncologists, pathologists, and intensivists among medical doctors. Other health care professionals, such as nurses and psychologists, but also biologists, computer scientists, nutritionists, and communication specialists have become relevant parts of the care process too. The high complexity of pancreatic disorders and the lack of private and public funding is a danger to the professional growth of these individuals and ultimately to the cure of pancreatic disorders (1).

Traditionally, education and research on the exocrine pancreas has received little attention from the medical community and limited economic support from the pharmaceutical industry. In Europe, a multidisciplinary team of professionals founded the European Pancreatic Club (EPC) in 1965, the first scientific society concerned with the study of the pancreas (2). Although the EPC served as a meeting point for consolidated pancreatologists, Pancreas 2000, an educational program established in 1999, on promoting education and research among young health care professionals. Pancreas 2000 is promoted by the Karolinska Intitutet (3) and receives support from the EPC and the United European Gastroenterology (UEG) and industry.

Pancreas 2000 was designed and has been continuously evolving over more than 20 years (3). Pancreas 2000 is a 2-year course involving 5 face-to-face meetings of 3 days. The students or mentees come from all over Europe. The mentors are widely acknowledged leaders in the study of pancreatic disorders, with the responsibility of helping mentees develop three skills, including knowledge of pancreatology, scientific thinking, and group leadership (3). A fourth result is the creation of a solid network of peers among these young professionals that is of help during their careers. To apply to Pancreas 2000, the students propose protocols for potential research studies. The best feasible protocols proposed by the admitted students then become research projects for a group of four to six mentees, led by two mentors. Mentees learn how to develop the research through detailed methodology and are in charge of determining the authorship rules, the development of the study, and writing the manuscript. The role of mentors is to guide the mentees, not to lead the studies, helping the younger colleagues to advance in skill development. During the different courses, the mentees receive sessions on different aspects of pancreatology by renowned experts in the field, and group leadership education sessions led by a professional coach. With more than 200 graduates in the last 21 years and dozens of scientific articles published, including pivotal studies on different topics in pancreatology (4–6). Pancreas 2000 has been a great success in terms of science and education. Most of the articles in this Frontiers issue on “Hot Topics in Pancreatology from Europe-2020” are protocols from the ninth course of the Pancreas 2000 initiative. Other studies from prominent researchers or research consortia complete the issue.

The topics range from studies investigating the clinical course of acute pancreatitis to protocols on pancreatic cancer, autoimmune pancreatitis, and ampullary neoplasms.

These topics reflect the whole spectrum of pancreatic disorders and highlight their relevance as a growing burden worldwide. The incidence of all pancreatic neoplasms is increasing, with pancreatic ductal adenocarcinoma (PDAC) projected to become the second leading cause of cancer-related death in most countries. Acute pancreatitis (AP) is always one of the most common causes of emergency room access among digestive disorders.

In addition, some less common types of tumors, such as pancreatic neuroendocrine neoplasms (pNEN) (Pulvirenti et al.) and ampullary tumors (AT) (Hollenbach et al.) have also shown an increased incidence. There are several shortcomings in the knowledge of these pancreatic disorders. Evidence on the clinical care of AP is limited (Lanzillotta et al.) and prospective studies or RCTs are difficult to perform [Bolado et al.; Cárdenas-Jaén et al.; (7)]. PDAC is a deadly disease and there is a desperate need for studies on its prevention, early diagnosis, and treatment (Hain et al.; Ronellenfitsch et al.). There are aspects of disease care that are under-investigated, such as nutritional (Kiriukova et al.) and psychological status (Consolandi et al.) and tumor biology is poorly understood [Zhang et al.; (8)]. Among the less common diseases, pNENs are increasingly diagnosed incidentally (9). This poses the relevant question of how to treat small incidental pNEN that may never progress but that still pose a threat to the life expectancy of patients. This critical aspect will be investigated by several studies presented in this issue (Tanno et al.; Pea et al.; Partelli et al.).

The education of selected, highly motivated, young researchers and the creation of networks and consortiums, together with a better understanding of complex biological phenomena, are key elements with great potential in future research in pancreatology.

The present decade is the one in which we will undertake a new odyssey toward “where we have never been” in the field of pancreatology, a journey from which we we cannot look back (10).

## Author Contributions

All authors listed have made a substantial, direct and intellectual contribution to the work, and approved it for publication.

## Conflict of Interest

The authors declare that the research was conducted in the absence of any commercial or financial relationships that could be construed as a potential conflict of interest. The handling editor declared a past co-authorship with the author Ed-M.

## Publisher's Note

All claims expressed in this article are solely those of the authors and do not necessarily represent those of their affiliated organizations, or those of the publisher, the editors and the reviewers. Any product that may be evaluated in this article, or claim that may be made by its manufacturer, is not guaranteed or endorsed by the publisher.
